# Supplementary material for: Cognitive Reserve in Early Manifest Huntington Disease Patients: Leisure Time Is Associated with Lower Cognitive and Functional Impairment
Source: J Pers Med. 2022 Jan 3;12(1):36. doi: 10.3390/jpm12010036 (PMC8777615; doi:10.3390/jpm12010036)
Supplement: Supplementary file 1 [file jpm-12-00036-s001.zip › jpm-1516278-supplementary.pdf]

Table S1: CAG expansion in our study sample

No.	Subject ID	Enroll-HD Centres	CAG expansion
1	HD-3	LIRH Foundation Rome	44
2	HD-4	LIRH Foundation Rome	46
3	HD-5	LIRH Foundation Rome	46
4	HD-6	LIRH Foundation Rome	43
5	HD-7	LIRH Foundation Rome	48
6	HD-9	LIRH Foundation Rome	43
7	HD-13	LIRH Foundation Rome	49
8	HD-14	LIRH Foundation Rome	44
9	HD-15	LIRH Foundation Rome	43
10	HD-16	LIRH Foundation Rome	42
11	HD-17	LIRH Foundation Rome	45
12	HD-19	LIRH Foundation Rome	44
13	HD-21	LIRH Foundation Rome	41
14	HD-22	LIRH Foundation Rome	41
15	HD-24	LIRH Foundation Rome	42
16	HD-25	LIRH Foundation Rome	42
17	HD-27	LIRH Foundation Rome	43
18	HD-28	LIRH Foundation Rome	46
19	HD-29	LIRH Foundation Rome	41
20	HD-32	LIRH Foundation Rome	43
21	HD-33	LIRH Foundation Rome	43
22	HD-34	LIRH Foundation Rome	49
23	HD-36	LIRH Foundation Rome	44
24	HD-37	LIRH Foundation Rome	44
25	HD-38	LIRH Foundation Rome	43
26	HD-39	LIRH Foundation Rome	42

27	HD-42	LIRH Foundation Rome	41
28	HD-45	LIRH Foundation Rome	45
29	HD-46	LIRH Foundation Rome	44
30	HD-50	LIRH Foundation Rome	41
31	HD-51	LIRH Foundation Rome	45
32	HD-52	LIRH Foundation Rome	42
33	HD-53	LIRH Foundation Rome	44
34	HD-56	LIRH Foundation Rome	47
35	HD-57	LIRH Foundation Rome	47
36	HD-58	LIRH Foundation Rome	45
37	HD-60	LIRH Foundation Rome	44
38	HD-62	LIRH Foundation Rome	47
39	HD-63	LIRH Foundation Rome	44
40	HD-65	LIRH Foundation Rome	41
41	HD-66	LIRH Foundation Rome	45
42	HD-67	LIRH Foundation Rome	45
43	HD-69	LIRH Foundation Rome	43
44	HD-71	LIRH Foundation Rome	44
45	HD-73	LIRH Foundation Rome	47
46	HD-74	LIRH Foundation Rome	48
47	HD-76	LIRH Foundation Rome	45
48	HD-77	LIRH Foundation Rome	44
49	HD-78	LIRH Foundation Rome	44
50	HD-79	LIRH Foundation Rome	42
51	HD-80	LIRH Foundation Rome	49
52	HD-81	LIRH Foundation Rome	43
53	HD-82	LIRH Foundation Rome	44
54	HD-83	LIRH Foundation Rome	40
55	HD-84	LIRH Foundation Rome	46
56	HD-85	LIRH Foundation Rome	41

57	HD-86	IRCCS Besta Milan	40
58	HD-87	IRCCS Besta Milan	40
59	HD-89	IRCCS Besta Milan	41
60	HD-91	IRCCS Besta Milan	43
61	HD-92	IRCCS Besta Milan	43
62	HD-93	IRCCS Besta Milan	40
63	HD-94	Sant'Andrea University Hospital	44
64	HD-95	Sant'Andrea University Hospital	42
65	HD-96	Sant'Andrea University Hospital	43
66	HD-97	Sant'Andrea University Hospital	42
67	HD-98	Sant'Andrea University Hospital	42
68	HD-99	Sant'Andrea University Hospital	44
69	HD-100	Sant'Andrea University Hospital	45
70	HD-101	Sant'Andrea University Hospital	41
71	HD-102	Sant'Andrea University Hospital	45
72	HD-103	Sant'Andrea University Hospital	45
73	HD-104	Sant'Andrea University Hospital	42
74	HD-105	Sant'Andrea University Hospital	47
75	HD-106	Sant'Andrea University Hospital	40
Mean repeat number ($\pm$ SD)			43,7 $\pm$ 2.3

Table S2: Correlations between CAG expansions and clinical variables

CLINICAL VARIABLES	CAG expansion	
	r	*p*
CRIq_Edu	-0,348	**0,002**
CRIq_WA	-0,320	**0,005**
CRIq_LA	-0,219	0,059
CRIq_Tot	-0,372	**0,001**
∆TFC	-0,008	0,946
∆TMS	-0,088	0,454
cUHDRS_t0	-0,040	0,736
cUHDRS_t1	-0,035	0,764
cUHDRS_t2	-0,039	0,738

CRIq_Edu: Cognitive Reserve Index Educational; CRIq_WA: Cognitive Reserve Index Working activity; CRIq_LA: Cognitive Reserve Index Leisure activities; CRIq_Tot: Cognitive Reserve Index Total score; ∆TFC: the difference between the Total Functional Capacity score at t2 and t0; ∆TMS: the difference between the Total Motor Score at t2 and t0; cUHDRS_t0: composite Unified Huntington's Disease Rating Scale at baseline; cUHDRS_t1: composite Unified Huntington's Disease Rating Scale at 1-year follow-up; cUHDRS_t2: composite Unified Huntington's Disease Rating Scale at 2-year follow-up.

Table S3: Correlations between CAG expansions and cognitive variables at three timepoints

COGNITIVE TEST		CAG expansion	
		r	*p*
BASELINE	MMSE	-0,020	0,865
	SDMT	0,054	0,647
	VFT	-0,102	0,382
	SCR	-0,004	0,974
	SWR	-0,018	0,876
I follow-up	MMSE	0,030	0,797
	SDMT	0,051	0,665
	VFT	-0,027	0,817
	SCR	0,006	0,963
	SWR	-0,019	0,874
II follow-up	MMSE	-0,1	0,397
	SDMT	-0,013	0,914
	VFT	-0,126	0,280
	SCR	0,052	0,655
	SWR	-0,026	0,824

MMSE: Mini Mental State Examination; SDMT: Symbol Digit Modality Test; VFT: Categorical Verbal Fluency Test; SCR: Stroop Color Reading Test; SWR: Stroop Word Reading Test.

Table S4.1: Clinical and cognitive scores in impaired and normal cognitive reserve total index groups.

		CRIq_Tot impaired Group (n=32) Mean ± SE	CRIq_Tot normal Group (n=43) Mean ± SE	p
Clinical variables	Δ_TFC	-1.69 ± 0.2	-1.33 ± 0.23	0.311
	Δ_TMS	10.5 ± 1.39	7.44 ± 1.48	0.149
	cUHDRS_t0	73.24 ± 8.5	85.27 ± 7.47	0.293
	cUHDRS_t1	62.84 ± 8.84	77.55 ± 8.14	0.229
	cUHDRS_t2	49.96 ± 9.72	69.15 ± 9.20	0.162
Cognitive variables - *Baseline*	MMSE	25.53 ± 0.5	27.01 ± 0.4	0.069
	SDMT	23.03 ± 2.26	26.55 ± 2.06	0.257
	VFT	13.4 ± 1.01	14.37 ± 0.73	0.431
	SCR	47.21 ± 2.86	50.69 ± 2.81	0.399
	SWR	65 ± 4.1	73.39 ± 3.76	0.140
Cognitive variables - *1 year follow-up*	MMSE	24.87 ± 0.66	26.04 ± 0.48	0.064
	SDMT	22.41 ± 2.37	25.64 ± 2.09	0.314
	VFT	12.31 ± 1.15	14.53 ± 0.77	0.101
	SCR	43.25 ± 2.62	49.23 ± 2.99	0.153
	SWR	61.37 ± 3.91	70.67 ± 3.96	0.107
Cognitive variables - *2 years follow-up*	MMSE	24.16 ± 0.73	26.04 ± 0.45	0.067
	SDMT	21.28 ± 2.06	24.98 ± 2.35	0.261
	VFT	11.65 ± 0.87	13.18 ± 0.71	0.176
	SCR	42.46 ± 3.54	47.23 ± 3.1	0.316
	SWR	55.66 ± 4.46	67.63 ± 4.19	0.067

Table S4.2: Clinical and cognitive scores in impaired and normal cognitive reserve education groups

		**CRIq_Edu impaired Group (n=14)** Mean ± SE	**CRIq_Edu normal Group (n=61)** Mean ± SE	**p**
**Clinical variables**	**Δ_TFC**	-1.64 ± 0.34	-1.44 ± 0.20	0.659
	**Δ_TMS**	9.14 ± 1.69	8.66 ± 1.22	0.857
	**cUHDRS_t0**	77.07 ± 15.01	80.848 ± 6.05	0.796
	**cUHDRS_t1**	73.14 ± 15.58	70.848 ± 6.55	0.883
	**cUHDRS_t2**	61.28 ± 16.56	60.898 ± 7.46	0.982
**Cognitive variables** - *Baseline*	**MMSE**	25.84 ± 0.91	26.59 ± 0.34	0.385
	**SDMT**	26.21 ± 4.1	24.78 ± 1.64	0.719
	**VFT**	13.5 ± 1.66	14.06 ± 0.64	0.717
	**SCR**	47.85 ± 4.3	49.52 ± 2.29	0.751
	**SWR**	66.5 ± 6.41	70.57 ± 3.13	0.575
**Cognitive variables** - *1 year follow-up*	**MMSE**	24.92 ± 1.22	25.96 ± 0.41	0.319
	**SDMT**	25.57 ± 4.12	23.96 ± 1.68	0.690
	**VFT**	11.92 ± 2.07	13.96 ± 0.67	0.238
	**SCR**	45.64 ± 4.39	46.91 ± 2.34	0.812
	**SWR**	67.42 ± 6.33	66.54 ± 3.21	0.904
**Cognitive variables** - *2 years follow-up*	**MMSE**	24.84 ± 1.21	25.623 ± 0.44	0.488
	**SDMT**	24.5 ± 3.89	23.15 ± 1.79	0.747
	**VFT**	11.85 ± 1.59	12.68 ± 0.58	0.565
	**SCR**	46.21 ± 4.55	44.96 ± 2.68	0.837
	**SWR**	63.21 ± 6.84	62.36 ± 3.53	0.916

Table S4.3: Clinical and cognitive scores in impaired and normal cognitive reserve working activity index groups.

		CRIq_WA impaired Group (n=29) Mean ± SE	CRIq_WA normal Group (n=46) Mean ± SE	p
Clinical variables	Δ_TFC	-1.52 ± 0.28	-1.46 ± 0.22	0.867
	Δ_TMS	10.14 ± 1.71	7.87 ± 1.31	0.293
	cUHDRS_t0	76.92 ± 9.31	82.17 ± 7.1	0.652
	cUHDRS_t1	69.2 ± 9.73	72.58 ± 7.74	0.786
	cUHDRS_t2	54.78 ± 10.93	64.86 ± 8.64	0.471
Cognitive variables - *Baseline*	MMSE	25.67 ± 0.55	26.95 ± 0.38	0.067
	SDMT	24.1 ± 2.59	25.65 ± 1.9	0.625
	VFT	14.89 ± 1.04	13.37 ± 0.72	0.219
	SCR	51.69 ± 3.49	47.65 ± 2.45	0.335
	SWR	68.44 ± 4.43	70.67 ± 3.65	0.702
Cognitive variables - *1 year follow-up*	MMSE	25.17 ± 0.73	26.15 ± 0.46	0.239
	SDMT	23.65 ± 2.59	24.68 ± 1.99	0.752
	VFT	13.31 ± 1.13	13.76 ± 0.83	0.746
	SCR	47.37 ± 3.34	46.23 ± 2.65	0.790
	SWR	66.17 ± 4.26	67.04 ± 3.83	0.883
Cognitive variables - *2 years follow-up*	MMSE	24.57 ± 0.78	26.04 ± 0.47	0.092
	SDMT	22.03 ± 2.63	24.26 ± 2.06	0.506
	VFT	12.31 ± 1	12.67 ± 0.66	0.753
	SCR	43.79 ± 3.75	46.08 ± 3	0.636
	SWR	60.03 ± 4.89	64.09 ± 4.07	0.531

CRIq_Tot: Cognitive Reserve Index Total score; CRIq_Edu: Cognitive Reserve Index Educational; CRIq_WA: Cognitive Reserve Index Working activity;
ΔTFC: the difference between the Total Functional Capacity score at t2 and t0; ΔTMS: the difference between the Total Motor Score at t2 and t0;
cUHDRS_t0: composite Unified Huntington's Disease Rating Scale at baseline; cUHDRS_t1: composite Unified Huntington's Disease Rating Scale at 1-year follow-up; cUHDRS_t2: composite Unified Huntington's Disease Rating Scale at 2-year follow-up.
MMSE: Mini Mental State Examination; SDMT: Symbol Digit Modality Test; VFT: Categorical Verbal Fluency Test; SCR: Stroop Color Reading Test; SWR: Stroop Word Reading Test.
SE: standard error; NS: not significant.
